# A randomized control trial to assess the impact of vitamin D supplementation compared to placebo on vascular stiffness in chronic kidney disease patients

**DOI:** 10.1186/1471-2261-14-156

**Published:** 2014-11-07

**Authors:** Adeera Levin, Taylor Perry, Prathibha De Zoysa, Mhairi K Sigrist, Karin Humphries, Mila Tang, Ognjenka Djurdjev

**Affiliations:** University of British Columbia, 1081 Burrard Street, Room 6010A, Vancouver, BC V6Z 1Y6 Canada; Providence Health Care Research Institute (PHCRI), St. Paul’s Hospital, 1081 Burrard Street, Room 302, Vancouver, BC V6Z 1Y6 Canada; Division of Nephrology Research - University of British Columbia/Providence Health Care, Vancouver, BC V6Z 1Y6 Canada; Providence Health Care (PHC), Division of Cardiology, Vancouver, BC V6Z 1Y6 Canada; BC Provincial Renal Agency (BCPRA), 700-1380 Burrard Street, Vancouver, BC V6Z 2H3 Canada

**Keywords:** Chronic kidney disease, Vitamin D, Vascular stiffness, PWV, Randomized protocol study

## Abstract

**Background:**

Vitamin D deficiency is associated with cardiovascular (CV) risk in multiple populations, including those with chronic kidney disease (CKD). The active form of the hormone (1,25 OH_2_D_3)_ binds to receptors in multiple organs. CKD patients are deficient in both 25 Vitamin D and 1,25 OH_2_D_3_. Clinical trial data demonstrating the benefits of vitamin D formulations are limited, and fail to show significant benefits on CV outcomes, and have compared different compounds, in various populations, and focused on a variety of outcomes. A understanding of the mechanism by which different vitamin D compounds confer CV protection in CKD is important for the design of future studies.

**Methods/Design:**

This 3 arm randomized prospective double-blinded placebo-controlled study examining the impact of calcitriol (1,25 OH_2_D_3_) and 25-hydroxyvitamin D3 supplementation compared to placebo on vascular stiffness, as measured by pulse wave velocity (PWV). Patients are enrolled from 2 tertiary care institutions if they meet inclusion criteria (stable estimated glomerular filtration rate (eGFR) between 15-45ml/min, <±5ml/min change in previous 6 months), on stable doses of renin-angiotensin aldosterone system blockade. For those already receiving vitamin D therapies, a 3 month washout period before randomization is mandatory. Treatment duration is 6 months; medications are given thrice weekly in fixed doses. The primary outcome measure is Vascular stiffness, measured non-invasively by pulse wave velocity (PWV). Other measurements include BP, kidney function and serial blood levels of biomarkers. The primary analysis will compare any vitamin D therapy versus placebo for the primary outcome defined as the change of PWV from baseline to 6 months. Analysis of covariance will be used to detect differences between vitamin D preparations in the magnitude of reduction in PWV.

**Discussion:**

This study is novel in that we are using a robust study design in CKD patients (not on dialysis) comparing placebo to different forms of vitamin D supplementation in fixed doses, irrespective of baseline values. We hope to demonstrate the biological mechanistic effect of vitamin D supplementation on vascular function in order for this information to be used in designing larger randomized controlled trials.

**Trial registration:**

Current Controlled Trials NCT01247311. Date of Registration: November 12, 2010.

**Electronic supplementary material:**

The online version of this article (doi:10.1186/1471-2261-14-156) contains supplementary material, which is available to authorized users.

## Background

Cardiovascular (CV) disease in chronic kidney disease (CKD) is highly prevalent and is associated with a significant increase in morbidity and mortality [1]. Vascular dysfunction and CV disease are caused by a combination of atherosclerotic processes (mediated by dyslipidemia and smoking) and arteriosclerotic processes such as aging, diabetes, vitamin D deficiencies, hyperphosphatemia and hyperparathyroidism (HPTH). The latter is associated with vascular stiffness, loss of collagen and elastin fibers [2]. CKD patients have both ‘conventional’ and non-conventional risk factors for CV disease; and high prevalence of left ventricular hypertrophy (LVH) has been noted. To date, conventional CV disease risk reduction agents and strategies (renal angiotensin aldosterone system (RAAS) antagonists, anti-platelet agents, lipid lowering agents) have either not been systematically evaluated in all CKD populations, or do not appear to mitigate CV disease risk to the extent that they are effective in non-CKD populations. Furthermore, strategies to address non-conventional risk factors (anemia, hyperphosphatemia) on outcomes have either shown no impact, harm, conflicting results, or have not been evaluated in a rigorous randomized control trial with clinically meaningful endpoints [3].

Vitamin D deficiency, (25 OHD_3_) is common in both CKD and general populations. It has been associated in animal and human studies with hypertension (HTN), diabetes, obesity and inflammation, and proteinuria. It is these associations that has led some to regard it as an important CV disease risk factor [4–6]. CKD patients have low levels of 25-hydroxyvitamin D (25 vitamin D), but also lack the physiological capacity to hydroxylate 25 vitamin D into its active form 1,25-dihydroxyvitamin D (1,25 vitamin D), which binds to the vitamin D receptors (VDR) found in blood vessels, the heart, muscles and kidneys. Animal studies describe the impact of VDR activators on inflammation in both kidneys and hearts. In addition, LVH has been noted, and reversed with supplementation [7]. [ Human studies have demonstrated reduction in proteinuria [8, 9] and some impact on changes in eGFR. These are small studies of limited duration. Thadhani et al. have found that treatment with paricalcitol (a VDR activator) in CKD patients reduces parathyroid hormone (PTH) levels within 4 weeks of commencing treatment versus a placebo [10], however, did not impact the primary outcome ( LV mass), but did reduce the number of CV disease-related hospitalizations over the trial period. Large cohort studies have also demonstrated reductions in 1,25 vitamin D and 25 vitamin D in CKD populations in association with rises in serum PTH values [11], at eGFR levels commencing around 50mL/min/1.73m^2^. While CKD patients may be able to hydroxylate 25 vitamin D outside the kidney, the relative effectiveness of these mechanisms have not been studied [2]. The prevailing theory is that the combined 25 OHD_3_ and 1,25 OH_2_D_3_ deficiencies seen in CKD do impact outcomes.

The association between 25 vitamin D deficiency and CV disease has been demonstrated in numerous populations [5, 12]. The active hormone modulates a variety of processes directly associated with arteriosclerotic changes via its interactions with specific biomarkers and inflammatory processes [13–17]. As a recent Cochrane review asserts, no prospective randomized controlled study to date have demonstrated the effect of vitamin D supplementation to reduce CV disease events [18]. Given the multiple reported effects of vitamin D on PTH, phosphate (PO_4_), blood pressure (BP), inflammation, insulin sensitivity and other factors, it is imperative to demonstrate a biological impact of vitamin D on vascular function, which could be directly in the causal pathway for CV disease [11, 15, 19, 20].

There are non invasive methods of studying vascular stiffness, the most used of which is Pulse wave velocity (PWV) using standardized validated equipment ( aSphygmoCor). This technique utilizes applanation tonometry to measure the velocity of the pulse wave between two points on the arterial tree [21]. Increased vascular stiffness ( higher PWV values) is associated with LVH, poor CV disease outcomes, and death in dialysis populations [22, 23]. The reproducibility and validity of this measurement has been demonstrated both in the general population and in those with kidney failure [22, 24, 25]. Utilizing PWV measurements, we will attempt to demonstrate changes in vascular stiffness with various forms of vitamin D supplementation in a non dialysis CKD population.

## Methods/Design

### Overarching objectives

The overarching objective of this study is to describe the impact of currently used formulations of vitamin D (25 vitamin D3 and calcitriol) on vascular stiffness in patients with CKD, eGFR levels between 15 and 45 mL/min/1.73m^2^. We hypothesize that supplementation with vitamin D analogues will result in greater reductions in PWV measurements than placebo when compared to baseline measurements.

### Study cohort

Subjects will be drawn from a cohort of CKD patients treated according to best practices at university based tertiary care centers in Vancouver. Following written informed consent, subjects who fulfill eligibility criteria at screening (Table 1) will be randomized (1:1:1) to receive treatment of 25 vitamin D3 at 5000IU three times a week, 1,25 vitamin D (calcitriol) at 0.5ug three times a week, or placebo.

**Table 1:** **Inclusion/exclusion criteria**

Inclusion criteria	Exclusion criteria
- Signed informed consent	- Refused informed consent
- eGFR between 15-45 ml/min	- Change of >5ml/min eGFR over the past 6 months
- Change of <5ml/min in eGFR over the past 6 months	- Planned transplant within 6 months
- Treated with maximal conventional CV disease risk reduction protocolized medications (ACEi or ARB)	- Likely to commence renal replacement therapy within 6 months after enrolment
- Currently receiving vitamin D therapies, but agreeing to a washout period of 3 months	- Terminal malignancies
	- Active infections
	- Active inflammatory diseases (SLE, vasculitis)

### Funding sources

The study is funded from a peer reviewed grant from Kidney foundation of Canada (KFoC) in collaboration with Pfizer. The study addresses specific interests of the KFoC, focusing on Canadians with CKD at risk for CVD, and identifying potential benefits of adjunct therapy in a complex condition.

### Statement of purpose

This physiological study will describe the impact of vitamin D therapies on a physiological measure related to CVD in CKD: vascular stiffness. Describing this mechanism would enhance the viability of testing longer term outcomes using these interventions.

*Primary questions:*

1. Does treatment with vitamin D analogues for a period of 6 months reduce PWV in CKD patients as compared to placebo?

*Secondary questions:*

1. Is there a difference between 1,25 vitamin D and 25 vitamin D formulations in the magnitude of reduction in PWV?
2. Does 6 month provision of 1,25 or 25 vitamin D lead to demonstrable reductions in BP (without changes in antihypertensive medication), or proteinuria (without change in ACEi/ARB therapy) relative to placebo?
3. What is the impact of 1,25 vitamin D and 25 vitamin D on blood levels of FGF-23, serum PTH, PO_4_ Ca, and CRP in this population? Is there a relationship between changes in vascular stiffness and changes in these biochemical/hormonal parameters?

### Rationale for the study

There is increasing evidence that vitamin D acting on multiple aspects of the physiological processes involved in the pathogenesis of CVD may be an important therapeutic strategy in CKD. The intellectual appeal of studying vitamin D supplementation in CKD has led to a number of proposals for studies of CV outcome in CKD, and some randomized control trials [3, 10, 26, 27]. While these studies have shown promising signals (reduction in proteinuria and reduction in CV hospitalization and events), no studies have addressed the mechanism of how vitamin D supplementation might impact these outcomes in humans, other than via reduction in LV mass (which was not shown). While both 1,25 vitamin D and 25 vitamin D formulations have been used in clinical studies to date, controversy continues as to the form of vitamin D best used in clinical practice to supplement deficiencies [16, 28, 29].

Recent meta-analyses and systematic reviews [15, 17, 26, 30–32] have described epidemiological associations with 25 vitamin D deficiencies and outcomes, and supplementation as impacting intermediate outcomes (proteinuria), but not mortality or time to end stage kidney failure. Quantification of the impact of vitamin D on vascular stiffness will permit estimates of effect size and stimulate further research into combining medication strategies.

### Interventions

We will prescribe fixed does of 5000IU 25 vitamin D and 0.5μg of 1,25 vitamin D, three times a week, (compatible with doses given in other studies, and at intervals used in many clinical practices) in order to maximally stimulate the circadian impact of the vitamin D therapy. A 6 month treatment period is chosen to ensure “biologically meaningful exposure” which should permit measurable changes in blood levels and vascular compliance, as well as mitigate the effects of sun exposure. Medications are supplied in oral suspension with identical look and taste (in almond oil), so that patients and providers are blinded.

### Duration of the study

Each patient will be followed for a period of 6 months post-randomization. Patients on existing vitamin D supplementation will have been followed for 9 months due to the additional 3 months of washout prior to randomization.

### Study design

**Figure 1:**
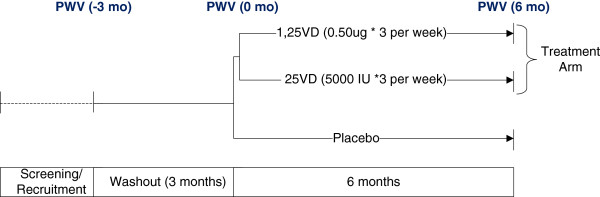
**The study plan depicts the timeline for the completion of the study.** Screening/recruitment includes the 3 month washout period. Vitamin D doses will be administered for 6 months allowing for the inclusion criteria. PWV will be measured at baseline (-3 months), post washout period (0 months) and post treatment (6 months) for both treatment arms and their respective placebos.

This will be a 3 arm prospective randomized double blind placebo controlled study of 128 stable CKD subjects examining the impact of vitamin D supplementation (1,25 vitamin D or 25 vitamin D formulations) compared to placebo on arterial stiffness and other parameters of vascular health. The study plan shown in Figure 1 will combine physiological experiments in a well characterized cohort of patients with CKD, and utilize observational study methods to test and explore additional related hypotheses.

### Sample size considerations

The study is powered to demonstrate differences in primary outcome between individual study subjects; the calculated sample size will be adequate to detect a clinically important effect of vitamin D on PWV (reduction of 1m/sec). The mean difference of 1m/s is clinically meaningful in that it has been demonstrated to confer a 34% increased risk of CV mortality in a cohort of CKD stage 5 patients on dialysis [33, 34]. A sample size of 105 (35 placebo arm and 70 treatment arm) without attrition will provide 85% power to demonstrate the mean PWV difference of 1m/s between arms, with 0.05 target alpha for a two sided test. Thus, recruitment and enrolment of 128 subjects will allow for ~15% attrition.

### Trial management

To ensure safety and data integrity, the single research office centrally located is responsible for the conduct at two sites within the city, both University teaching hospitals. The study will be overseen by independent Data Safety and Monitoring Board (DSMB) and also will review subject lab results and concomitant medications on a monthly basis. The DSMB will screen for the adverse events, including the possibility of hypervitaminosis, hypercalcaemia and hyperphosphatemia. In addition, random audits will be performed to assess data quality.

### Variables measured

PWV measurements, clinical and biological samples will be obtained at pre-washout, baseline, and post treatment (3 time points). Blood and urine samples will be obtained at each of the 3 time points to test for serum PTH, 1,25 vitamin D, 25 vitamin D, fibroblast growth factor-23 (FGF-23), PO_4_, calcium (Ca), C-reactive protein (CRP), urinalysis and random urine albumin-to-creatinine ratio (UACR). All samples will be run in an accredited laboratory using validated assays. BP will be done in duplicate and PWV will be done in triplicate at each visit using standardized BP cuffs and SphygmoCor, respectively [35]. Clinical and medication histories as well as rationale for specific biomarkers are listed in Additional file 1.

### Statistical analysis

The primary analysis will compare vitamin D (25 vitamin and 1,25 vitamin D) therapies versus placebo for the primary outcome defined as the change of PWV from baseline to 6 months by covariance analysis (ANCOVA); where the 6 month measurement of PWV will be the dependent variable, allocated treatment the independent variable, and baseline PWV the baseline covariate [29, 36, 37]. Analyses will be considered statistically significant if the p-value is <0.05 for allocated treatment. In case of violation of ANCOVA assumptions we will use a mixed model approach.

### Strengths and weaknesses

It is conceivable that the therapies as administered may not have an impact on our chosen outcomes of interest: vascular stiffness, BP or proteinuria (primary or secondary). The 6 month time period may not be sufficient exposure to demonstrate the effect of the vitamin D treatment, or the expected difference in PWV changes/reductions between the groups is different and thus the study may be underpowered. However, given that this study uniquely examines non dialysis advance stage CKD patients, using fixed dose drug administration schedules, physiological measurements and biochemical measurements serially over time, the study will add to the growing body of literature regarding the use of vitamin D supplementation in CKD population. If positive, it will inform future larger studies looking at additional measures such as LV mass, or function over time in relation to PWV, and will add to our understanding of the physiology of vascular changes in CKD. Given the current state of knowledge, and the plethora of studies, reviews, and meta-analysis that fail to consistently demonstrate a benefit to Vitamin D supplementation, there are no ethical issues conducting a placebo-controlled trial.

## Discussion

There is an accumulating body of evidence that vitamin D acting on multiple aspects of physiological processes involved in the pathogenesis of CVD and thus may be an important therapeutic strategy in CKD. Studies conducted to date have failed to demonstrate significant impact of hormone supplementation on outcomes. Cholecalciferol has been studied in 60 patients on hemodialysis, who were known to be 25 OHD_3_ deficient and did not show any change in PWV [38]. Other studies conducted in both dialysis and non dialysis CKD patients, also included were those with demonstrable deficiencies for limited time periods (8 weeks) showed changes in serum levels of 25 and 1, 25 Vitamin D levels [39–42]. Although more pronounced in non dialysis patients, other outcomes were not measured. Another study demonstrated that 25 OHD_3_ supplementation in post menopausal women did not reduce CV risk, when studied in a randomized control trial [43]. These studies were all relatively short term and focused on different questions than the ones we are attempting to answer in this study. We believe that attempting to establish a biological mechanistic effect of vitamin D supplementation on vascular function is necessary before further larger randomized controlled trials are undertaken. Given the purported differential ability of CKD patients to achieve appropriate concentrations of the active compound, the question of which supplementation is more appropriate, 25 vitamin D or 1,25 vitamin D in CKD remains controversial. The relationship of serum levels to disease, type of therapeutic supplementation (fixed dose or based on targeting serum levels) is unknown in CKD populations. Moreover, in various nephrology studies, attempts at targeting deficiencies or excesses of measured serum values in CKD patients in large randomized control trials has not met with success [44–47]. Thus, we have developed and designed a robust randomized control trial to attempt to address deficiencies in the literature, and to better understand mechanisms and complex biological interactions in this patient group.

We expect to find vitamin D to have an impact on vascular stiffness and BP irrespective of serum vitamin D levels. The impact will be greatest on those with highest PWV. The data generated herein will permit the development of a set of phenotypes using clinical and biomarker data. Such that characterization of patients according to PWV, BP, FGF-23 and vitamin D levels will be possible, and results of treatment strategies will be examined within the phenotype category. This information will be significant for future enrolment in clinical trials. The biomarker analyses will aid in explaining any dissociation found between BP and vascular stiffness.

Before large randomized trials can be developed, these unanswered questions regarding the mechanism of disease require resolution to ensure pertinent study design. This is an ‘intermediate’ study which fully examines the physiological consequences of different vitamin D therapies in CKD populations. The findings will be used to understand physiological mechanisms, generate further hypotheses for testing, and ultimately contribute to evidence base studies which will inform clinical practice.

### Ethics

The study has been approved by the University of British Columbia and Providence Health Care Research Ethics Committee (Registration number: H10-01689).

## Electronic supplementary material

Additional file 1: **Timetable of study visits.** (PDF 12 KB)

## Abbreviations

CV: Cardiovascular
CKD: Chronic kidney disease
BP: Blood pressure
eGFR: Glomerular filtration rate
PWV: Pulse wave velocity
HPTH: Hyperparathyroidism
HTN: Hypertension
PTH: Parathyroid hormone
PO4: Phosphate
LVH: Left ventricular hypertrophy
DSMB: Data safety and monitoring board
FGF-23: Fibroblast growth factor-23
Ca: Calcium
CRP: C-reactive protein
UACR: Urine albumin-to-creatinine ratio
ANCOVA: Analysis of covariance.
