# Supplementary material for: A randomized control trial to assess the impact of vitamin D supplementation compared to placebo on vascular stiffness in chronic kidney disease patients
Source: BMC Cardiovasc Disord. 2014 Nov 7;14:156. doi: 10.1186/1471-2261-14-156 (PMC4230794; doi:10.1186/1471-2261-14-156)
Supplement: Supplementary file 1 — Additional file 1: Timetable of study visits. (PDF 12 KB) [file 12872_2014_798_MOESM1_ESM.pdf]

Additional file 1.

Appendix 1: Timetable of study visits

Assessment	Screening visit (-3 months)	Washout visit (0 months)	Treatment visit (6 months)
Demographics, clinical review, history	x		
Medication Review	x	x	x
Pulse Wave Velocity	x	x	x
Blood Pressure	x	x	x
Blood Sample: (PTH, 1,25 vitamin D, 25 vitamin D, FGF-23, PO_4, Ca, CRP)	x	x	x
Urine Sample: (UACR and 24 hr urine protein)	x	x	x

Rationale for specific biomarkers:
There are a number of biological markers, measured in the blood, which mark for specific processes that are either directly or indirectly associated with the vitamin D pathways of interest. It is beyond the scope of this paper to describe each of these in detail, however, extensive literature is available in defining the roles of Ca, PO_4, PTH, FGF-23, CRP and other factors which are either impacted by vitamin D deficiencies or mitigate vitamin D effects.

Abbreviations

CV: Cardiovascular; CKD: Chronic kidney disease; BP: Blood Pressure; eGFR: Glomerular Filtration Rate; PWV: Pulse wave velocity; HPTH: hyperparathyroidism; HTN: Hypertension; PTH: Parathyroid hormone; PO_4: phosphate; LVH: Left ventricular hypertrophy; DSMB: Data Safety and Monitoring Board; FGF-23: fibroblast growth factor-23; Ca: calcium; CRP: C-reactive protein; UACR: urine albumin-to-creatinine ratio; ANCOVA: Analysis of Covariance.

Author details

[1]University of British Columbia, 1081 Burrard Street, Room 6010A, Vancouver, BC V6Z 1Y6, Canada. [2,3,7]Providence Health Care Research Institute (PHCRI), St. Paul's Hospital, 1081 Burrard Street, Room 302, Vancouver, BC V6Z 1Y6. [4]Division of Nephrology Research - University of British Columbia/Providence Health Care, Vancouver, BC V6Z 1Y6. [5]Providence Health Care (PHC), Division of Cardiology, Vancouver, BC V6Z 1Y6. [6]BC Provincial Renal Agency (BCPRA) 700-1380 Burrard Street, Vancouver, BC V6Z 2H3.
